# How bilinguals refer to Mandarin throwing actions in English

**DOI:** 10.1177/13670069211022853

**Published:** 2021-06-04

**Authors:** Elena Nicoladis, Helena Hong Gao

**Affiliations:** University of Alberta, Canada; Nanyang Technological University, Singapore

**Keywords:** Thinking for speaking, conceptual overlap, near-synonyms, cross-linguistic differences, conceptual equivalence

## Abstract

**Aims and Objectives:**

In the present study, we tested how Mandarin-English bilinguals choose English words to refer to prototypical Mandarin throwing actions. Languages differ in how they refer to events. In Mandarin and English, words for throwing actions differ notably on a variety of dimensions so there are few perfect translation equivalents. In previous studies, when faced with the challenge of how to speak about such events, bilinguals sometimes use language-specific ways in each language, sometimes show convergence, sometimes use more general terms, and there are times when they can be quite creative.

**Design/Methodology:**

We showed video clips of six prototypical Mandarin throwing actions (corresponding to *rēng 扔, diū* 丢, *pāo 抛, tóu 投, shuāi 摔, shuǎi 甩*) to Mandarin-English bilinguals and English monolinguals. Participants labeled the actions and chose the English word most closely corresponding to the action. The bilinguals did the same in Mandarin.

**Findings/Conclusion:**

The results showed that the bilinguals chose many of the same words in English as English monolinguals did. However, the bilinguals differed from the monolinguals in two ways: (1) they tended to choose more different responses and (2) they referred to *diū* 丢 actions most often as *throw* rather than *lob* as the monolinguals did.

**Originality:**

These results suggest that bilinguals use a variety of strategies to refer to the not-easily-translatable.

Languages differ in how they refer to events (Narasimhan et al., 2012; Pavlenko, 2014; Talmy, 1985). In some languages, emotions are states of mind and, in others, actions (Apresjan, 1997; Pavlenko, 2008). Some languages require tense marking, others mark only for aspect (Chen & Shirai, 2010; Sugaya & Shirai, 2009). Some languages have grammatical gender for every noun, others do not (Bender et al., 2011; Vigliocco et al., 2005). Some researchers have argued that the structure of language could influence how speakers of that language think (Casasanto, 2016; Filipović, 2013; Lucy, 2016; Matlock et al., 2005; Whorf, 1956). At the lexical level, one way in which language might influence thought is in guiding attention to particular aspects of the world (Green, 1998). For example, in Spanish, choosing an appropriate verb to describe the motion of a figure critically involves attending to whether the figure has crossed a boundary or not (Cadierno & Ruiz, 2006; Hohenstein et al., 2006; Slobin, 1996; Slobin & Hoiting, 1994; see Pavlenko, 2014, for a more in-depth review). To date, the research evidence for linguistic relativity is mixed, with some research finding supporting evidence (Athanasopoulos, 2009; Aveledo & Athanasopoulos, 2016; Roberson et al., 2005) and other research not (January & Kako, 2007; Li & Gleitman, 2002; Samuel et al., 2019). Thus, the more pertinent questions about language and thought have become in what circumstances and how does language influence thought (Casasanto, 2016).

To the extent that language structure influences thinking, bilinguals are faced with the challenge of thinking for speaking in each of their languages. There is evidence suggesting that bilinguals use a variety of strategies to cope with structural (and conceptual) differences between their languages (Pavlenko, 2000, 2009, 2014). Strategies refer to the methods or techniques of carrying out a task (Lee et al., 1995). In some cases of language differences, bilinguals show language-specific language and thought patterns in both of their languages (Alvarado & Jameson, 2011; Pavlenko, 2010; Pavlenko & Volynsky, 2015). For example, Pavlenko (2002, 2008) found that Russian-English bilinguals speak about emotions using Russian metaphors of emotions as actions in Russian and English metaphors of emotions as states in English. In other cases, bilinguals may use the language and thought patterns of one language in both of their languages (Odlin, 2005), or uni-directional transfer. For example, Malt and Sloman (2003) found that second language (L2) users of English showed patterns of naming common household objects consistent with their first language (L1) even after years of residence in the USA. In other cases still, bilinguals may show convergence (or bidirectional transfer), relying on language and thought patterns that are influenced by both of their languages (Ameel et al., 2005). In cases of convergence, bilinguals differ from monolinguals in both of their languages. For example, Spanish-English bilingual adults show some signs of convergence in speaking about motion events using more verbs referring to the manner of motion in Spanish than monolinguals and slightly more verbs referring to the path of motion in English than monolinguals (Hohenstein et al., 2006). These patterns suggest that the bilinguals are using a more Spanish-typical verb choice in English and a more English-typical verb choice in Spanish relative to monolinguals (see Pavlenko, 2014, for a review).

While bilinguals sometimes show language-specific patterns, sometimes uni-directional transfer, and sometimes convergence, it is not entirely clear which strategies bilinguals use with which linguistic structures (Hernandez et al., 2005; Malt et al., 2015). Part of the challenge in identifying when bilinguals use which strategies is due to methodological choices among psycholinguists: Pavlenko (2009) argued that when testing how bilinguals’ two languages were related in processing, psycholinguists traditionally avoided the inclusion of words that differ conceptually across languages. Where there is conceptual equivalence across languages, bilinguals are likely to rely on the same concept for both languages (Pavlenko, 2009, 2014).

Nevertheless, researchers have identified at least three word/concept-level variables that might affect bilinguals’ strategy choice: the degree of abstractness of the concept, the degree of complexity in the conceptual domain under study, and the degree of overlap of the concepts across the two languages (Gullberg, 2011; Pavlenko, 2008, 2014). In terms of abstractness, research has shown that convergence is more likely with concrete words than abstract words and with nouns more than verbs (Van Hell & De Groot, 1998).

Another variable that can affect bilinguals’ choice of strategies in processing words across languages is the degree of complexity in the conceptual domain. Some previous research has shown that L2 learners have particular difficulties in naming when their L2 is highly complex (i.e., makes many semantic/conceptual distinctions) relative to the L1 (Gullberg, 2011). However, Ibarretxe-Antuñano et al. (2016) found some counterevidence in a study of placement events among Danish-Spanish and Spanish-Danish bilinguals. Both Danish and Spanish have a high degree of complexity when referring to placement events. Yet, the bilinguals showed many differences from native speakers of the two languages in referring to placement events. The bilinguals used some of the same verbs, but also a larger number of different verbs than L1 speakers. These results suggest that the bilinguals retained the complexity of the semantic categories in both languages, but did so differently from monolinguals.

As for the degree of overlap, when there is no overlap in the concepts across languages, bilinguals are found to have different strategies in coping with the missing concepts. In some situations, bilinguals may select a generic word to refer to the concept. For example, studies (Gao, 2010, 2015) show that both English-Chinese bilingual children and adults tended to overuse the generic classifier *ge*, while Chinese-Swedish bilingual children tended to create their own classifiers. Some languages (e.g., Chinese, Japanese, or Thai) consistently use classifiers to refer to measure words (e.g., in English, *pair* in a *pair of shoes* could be considered a classifier). English is considered a non-classifier language because there are few classifiers and they are rarely obligatory. In other situations, bilinguals may code-switch, as is often observed in bilingual speakers’ communication in Singapore, or explain what the concept means and/or the conditions under which the concept occurs (Barbosa et al., 2017; Panayiotou, 2004).

In cases of partial overlap, several lexical/conceptual strategies are possible, including nesting, splitting, and overlap (Pavlenko, 2008). For example, Pavlenko and Driagina (2007) found that when speaking English, Russian-English bilinguals referred to the Russian concept of s*erdit'sia* as *angry*, although the latter is a broader concept than the former. Similarly, bilingual speakers whose L1 is a non-classifier language (e.g., English, French, or German) and L2 a classifier language are often found to make mismatches between a classifier and a noun if the classifier is not also a measure word whose equivalent exists in their L1, such as *pair* in a *pair of shoes* and *liter* in a *liter of milk* (Gao, 2008, 2010, 2015; Quek & Gao, 2011).

In the present study, we test how Mandarin-English bilinguals conceptualize nonequivalent verbs in L2 English. Specifically, this study targets verbs that are near-synonyms for *throw* (which itself is an example of a placement verb; see Narasimhan et al., 2012). Throw verbs depicting throw actions belong to a sub-class of physical action verbs characterized with “hand contact as a pre-condition” (Gao, 2001, p. 25). That is, to perform an action such as *throw, cast*, or *toss*, one first needs to pick up an object. This pick-up action is a pre-action or preparation action that is a covert lexical semantic property that is cognitively perceived and can help language learners to identify the type of throwing, which is to discriminate it from other types of throwing, or other synonymous *throw* verbs. The basic conceptual knowledge that forms the semantics of *throw* verbs also includes the information of body part, the hand(s) in this case, the throw action manner, the degree of force applied, the motion direction of the hand(s), and a causative result to the object thrown. All the words that fall into the class of *throw* verbs in a language share this information as semantic properties, but due to the fact that the manner, the degree of force applied, the motion direction of the hand(s), and the causative result to the object thrown depicted by each of the *throw* verbs are different, the class members are not perfect synonyms but near-synonyms.

Synonyms are words that have the same or nearly the same meaning in a language, such as *truck* and *lorry, small* and *little*. Perfect synonyms, such as *couch* and *sofa* are rare, but near-synonyms that are more or less similar, but not identical, in meaning are numerous (De Jonge, 1993; Lyons, 1995). Words that can be grouped into one class are generally near-synonyms because they are similar in meaning, but also differ in some semantic components, such as force or directionality (see also Ibarretxe-Antuñano et al., 2016) . They may also be similar in lexicalization patterning or event structure (Divjak, 2006). For example, all *throw* verbs require a human agent to be the subject of the sentence and the event structure of an agent throwing an object.

Mastering near-synonyms is challenging for L2 learners (Ahmadian & Darabi, 2012). Dictionaries (including thesauri) might not be very helpful to L2 learners, since even the best ones contain only some simple descriptions of the nuances of near-synonyms. For this reason, dictionaries generally cannot help learners in the acquisition of the conceptual knowledge that is embedded within similar words that have subtle differences.

Previous studies on monolingual and bilingual speakers’ acquisition of *throw* verbs in Mandarin and English show that both Mandarin and English have rich vocabularies to refer to throwing events (Gao et al., 2016; Wang & Gao, 2016). Other than the most general terms for throwing (i.e., *throw* in English and *rēng* 扔 in Mandarin), the near-synonyms in the two languages do not map neatly onto each other. Wang and Gao (2016) asked Mandarin- and English-speaking participants to act out the throwing actions corresponding to the most frequent throw verbs in Mandarin (i.e., *rēng* 扔, *diū 丢, pāo* 抛, *tóu* 投, *shuāI* 摔, *shuǎI* 甩) and in English (i.e., *throw, fling, chuck, cast, toss, hurl*). The results showed that, within a language, the verbs differed from each other in terms of force, directionality (both vertical and horizontal), initial arm position, and initial hand height (Ibarretxe-Antuñano [2012, 2016] have identified some similar characteristics as the basis for cross-linguistic differences in placement and removal verbs in other languages). Based on these dimensions, the Mandarin verbs tended to be highly dissimilar from each other, while the English verbs showed a narrower range of use of these dimensions. Of particular relevance for this study, there were very few perfect translation equivalents in Mandarin and English (cf. *toss- diū 丢*).

## This study

The purpose of the present study was to test the strategies Mandarin-English bilinguals use when referring to typical Mandarin throwing actions in English (following the methodology in Wang & Gao, 2016). While both Mandarin and English have rich vocabularies to refer to throwing events, there is little to no exact overlap in the words referring to these events in the two languages (Gao et al., 2016; Wang & Gao, 2016).

While some previous studies have found that bilinguals use target-like words in both languages (Pavlenko, 2010), we predicted that Mandarin-English bilinguals would differ from English monolinguals in labeling the events, because of the influence of the Mandarin concepts. How then might bilinguals select words in English to refer to Mandarin concepts? We sought evidence for the use of three strategies that have been documented previously in other similar studies.

1) Bilinguals might use the more generic *throw* for all verbs (see similar results in Gao, 2010, 2015). This strategy could mean that the bilinguals are in the process of learning the English near-synonyms as they are used in English.
2) Bilinguals might add modifiers to *throw* to refer to the salient conceptual characteristics of the Mandarin verbs (cf. Panayiotou, 2004). Use of this strategy would suggest that the bilinguals are conceptualizing the actions consistently with the Mandarin concepts and are searching for how to convey the important aspects of those concepts in English.
3) Bilinguals’ choices might be related to the degree of overlap in Mandarin and in English. Specifically, they might use similar words as monolinguals for words with close equivalents, specifically for *rēng* (i.e., *throw*) and *diū* (i.e., *toss*), since these actions are closely equivalent in the two languages (Wang & Gao, 2016). Where the concepts are only partially equivalent (i.e., *pāo, tóu, shuāi, shuǎi*), the bilinguals might show more varied responses than monolinguals (as found in Ibarretxe-Antuñano et al., 2016) and/or select words in English with some similar characteristics.

We used two tasks to assess the participants’ choices: a labeling task and a word-choice task. The strategies used by the participants in the two tasks might differ because of the task demands. As the pre-condition was to be able to have access to and differentiate a number of synonymous words to label the actions, they might be particularly likely to use the more generic term or add modifiers to *throw* – a coping strategy that is often found in bilingual learners in tasks in which lexical retrieval is challenging. In contrast, in the word-choice task, they might be more affected by the degree of overlap with Mandarin and English words.

The bilinguals also performed both the labeling and the word-choice task for the very same throwing actions in Mandarin. We expected the bilinguals to choose the target word, as has been found for Mandarin monolinguals in Wang and Gao’s (2016) study.

## Method

Forty Mandarin-English sequential bilinguals (22 females, 18 males) and 33 English monolinguals (25 females, eight males) participated in this study. All of the participants were undergraduate university students at an English-language Canadian university. The monolinguals averaged 18.8 years (SD = 1.0) of age. The bilingual participants averaged 20.6 years (SD = 1.7) of age and spoke Mandarin as a L1. All but one of the bilingual participants was born in Asia (mainland China, Taiwan, or Malaysia) and averaged 4.0 years (SD = 2.7) living in a predominantly English-speaking part of the world. The one remaining participant was born and lived her entire life in Canada. Her data were not outliers on any of the analyses.

We did not systematically measure the bilinguals’ English proficiency. Instead, we asked which language they spoke better. All participants except the Canadian-born participant reported that they spoke Mandarin much better than English. The Canadian-born participant reported that she spoke English better than Mandarin. As all the bilinguals spoke Mandarin with their family members at home and with their Chinese friends in social gathering, we assumed that their Mandarin competence was still at the mother tongue level, even though they were living and studying in Canada using English as the media for education.

### Materials

We presented participants with 12 video clips of prototypical Mandarin throwing actions, two different clips representing each of six actions. These were the same videos as used by Wang and Gao (2016). The thrower always threw with the right hand. The object thrown for all throwing actions was a small black-and-white plastic cube (approximately 5 centimeters in height, width, and depth), which was unnamable by the participants. Wang and Gao (2016) found that the near-synonyms in Mandarin differed in terms of force, how high the hand was when the action started, whether the arm was bent or straight, the horizontal direction, and the vertical direction. Table 1 summarizes some of the salient characteristics of the six actions. It is important to keep in mind that these are crude classifications; a more careful description of these verbs would show that there are few perfect translations of these near-synonyms (see Wang & Gao, 2016, for more complete descriptions).

**Table 1.** Some salient characteristics of Mandarin and English near-synonyms for throwing actions.

	Force	Hand	Arm	Horizontal direction	Vertical direction
rēng 扔	Medium	Medium	Straight	Forward	Upward
diū 丢	Light	Low	Straight	Forward	Upward
pāo 抛	Medium	Medium	Bent	Forward	Upward
shuǎi 甩	Strong	Medium	Bent	Forward	Downward
shuāi 摔	Strong	High	Bent	Forward	Downward
tóu 投	Medium	High	Bent	Sideways	Upward
throw	Medium	High	Bent	Forward	Upward
fling	Medium	Medium	Bent	Forward	Upward
chuck	Medium	Medium	Bent	Forward	Upward
cast	Medium	Medium	Bent	Forward	Upward
hurl	Strong	High	Bent	Forward	Upward
toss	Medium	Low	Straight	Forward	Upward

*Note*. From Wang and Gao (2016).

For the labeling task, participants were provided with a sheet of paper with 12 lines to write down words to refer to the actions. For the forced-choice task, participants were asked to choose which verb best corresponded with the action. In Mandarin, they could choose from *rēng* 扔, *diū 丢, pāo 抛, shuǎI 甩, shuāI 摔*, or *tóu 投* . In English, they could choose from *throw, fling, cast, hurl, lob, chuck*, or *sling*. These verbs are among the most frequent words to refer to throwing in each language (Wang & Gao, 2016).

### Procedure

Bilingual participants performed these tasks in both Mandarin and English during two different testing sessions, one in each language. The order of the languages was counterbalanced across participants. The two language sessions were held on different days, usually separated by about a week, and run by a native speaker of the target language of the session. Due to an experimenter error, six bilinguals were not tested in Mandarin. So the Mandarin data only include 34 participants.

Within a language session, participants performed the labeling task first: the 12 video clips were shown in a random order to each participant. The instructions for the labeling task were, “For each video clip, fill in the blank with a word that describes the action the man is doing.” They wrote down a label for the action and then advanced to the next video only when they had written down a label. After labeling all 12 actions, they watched the video clips (in the same order as for the labeling task) and circled the choice that best corresponded to the action. The instructions for the forced-choice task were, “For each video clip, choose the best word to describe the action the man is doing.”

### Coding and analysis

As we were interested in the way the individual Mandarin actions influenced participants’ responses, all analyses were conducted across items rather than across participants. In the present study, we included in the analyses only the participants’ first response for a particular action.

For the labeling task, we expected many participants to label many of the actions with *throw*, as the most general way of referring to throwing. Of particular interest in that task is when participants were sufficiently dissatisfied with *throw* to search for ways to modify *throw* or to use a near-synonym. We therefore present the percentage of labels of *throw* alone for each verb and the percentage of *throw* with modifying words (such as *up* or *hard*), as well as the most frequent choice. To calculate the percentages of *throw*, we classified any instances of modified *throw* as not-*throw*.

For the forced-choice task, for each action, we compared the responses of bilinguals and monolinguals using chi-squares on the number of responses. As chi-squares are sensitive to the unit of measurement, all of these analyses were performed on the numbers of participants. For ease of interpretation across groups of different sizes, however, in the results section, we present the graphs in percentages. In order to meet the assumption of chi-square analyses that all cells have at least five instances, to compare the bilinguals and the monolinguals, the bilinguals’ most frequent choice was compared to all responses that were not that choice, as well as to the monolinguals’ choice for those same words. For pāo 抛, for example, the most frequent English choice about the bilinguals was *fling* (*N* = 10) and 30 participants chose something other than *fling*. Six monolinguals also chose *fling* as the response for pāo 抛 (so 27 did not). The expected values were calculated as the cross-products of the rows and columns. When the two language groups were compared, a significant chi-square suggests that whether the participants were bilingual or monolingual made a difference in their choice.

A power analysis revealed that the necessary sample size to achieve power of 80% with medium effect size was 88. Note that in some of the analyses presented below, the participants did not make enough responses to reach that target. Those analyses should be interpreted with caution.

## Results

### Mandarin

Table 2 summarizes the bilingual participants’ labels in Mandarin for the Mandarin verbs (see the Appendix for a complete listing of responses). The target verb was among the most frequent responses for all actions except diū 丢. The most frequent label for that action was the generic *rēng* 扔. Also, note that the participants only occasionally added modifiers to *rēng* 扔. In the few instances in which they did so, the modifiers specified the direction of the action.

**Table 2.** Summary of bilinguals’ labels of typical Mandarin throwing actions in Mandarin.

	Most frequent label (%)	rēng 扔+ (%)	Words modifying rēng 扔
rēng 扔	rēng 扔 (44.1)	0	n/a
diū 丢	rēng 扔 (41.1)	2.9	向左 (direction)
pāo 抛	pāo 抛 (85.3)	0	n/a
shuǎi 甩	shuǎi 甩/diū 丢 (23.5)	5.8	向左下 (direction)
shuāi 摔	shuāi 摔 (44.1)	8.8	向下 (direction)
tóu 投	tóu 投 (41.1)	2.9	向上 (direction)

Table 3 summarizes what the bilingual participants chose in Mandarin in the forced-choice task for the Mandarin verbs. The most frequent choice for all the actions was the target word. However, for diū 丢, both *diū* 丢 and the generic *rēng* 扔 were chosen at roughly equivalent rates.

**Table 3.** Summary of bilinguals’ Mandarin responses on the forced-choice task.

	rēng 扔 (%)	Target (%)
rēng 扔	38.2	38.2
diū 丢	32.4	36.8
pāo 抛	1.5	94.1
shuǎi 甩	7.4	70.6
shuāi 摔	4.4	94.1
tóu 投	10.3	61.8

In summary, on both tasks in Mandarin, the bilinguals tended to choose the target verb, as predicted. The similarity of *diū* 丢 and *rēng* 扔 could affect the participants’ responses in English.

### English: Labeling

Table 4 summarizes the English monolinguals’ responses for labeling the actions. As can be seen in Table 4, the majority of English monolinguals used *throw* to label rēng 扔. For the other actions, they often used other words. Comparing all the actions to the proportion of *throw* expected for rēng 扔 yielded a significant chi-square, χ^2^ (*N* = 165, *df* = 4) = 69.89, *p* < .001. This result suggests that the monolinguals were labeling all the other actions significantly differently from rēng 扔. Table 4 also summarizes the most frequent label the monolinguals gave for each action. For both diū 丢 and pāo 抛, the majority labeled *toss*. For the remaining actions, the most frequent label was *throw*, but that label represents a minority of their responses. Notably, for shuǎi 甩, shuāi 摔, and tóu 投, the monolinguals seemed to avoid *throw*. Individual speakers labeled the actions quite differently (see the Appendix).

**Table 4.** Summary of monolinguals’ (M) and bilinguals’ (B) labels of typical Mandarin throwing actions in English.

		Most frequent label (%)	Throw + (%)	Words modifying throw
rēng 扔	M	Throw (78.8)	3.0	Overhand
	B	Throw (62.5)	10.0	Away, forward, out
diū 丢	M	Toss (72.7)	0	n/a
	B	Throw (60.0)	5.0	Down, out
pāo 抛	M	Toss (66.7)	3.0	Underhand
	B	Throw (50.0)	7.5	High, up, upward
shuǎi 甩	M	Throw (39.4)	0	n/a
	B	Throw (47.5)	10.0	Away, left, horizontally
shuāi 摔	M	Throw (24.2)	3.0	Forcefully
	B	Throw (22.5)	5.0	Down, hard
tóu 投	M	Throw (33.3)	3.0	Overhand
	B	Throw (42.5)	5.0	Down, high

Table 4 also summarizes the bilinguals’ responses for labeling the actions. The most frequent label for all actions was *throw*; however, this was the majority response for only rēng 扔 and diū 丢. A chi-square revealed a significant difference in the responses to non-rēng 扔 actions relative to rēng 扔, χ^2^ (*N* = 200, *df* = 4) = 21.76, *p* < .001. Like the monolinguals, for shuǎI 甩, shuāI 摔, and tóu 投, the bilinguals tended to avoid *throw*.

As can be seen in Table 4, for all actions, the bilinguals more frequently than the monolinguals added on modifying words to *throw* indicating directionality (e.g., *down* and *out* for diū 丢) or force (e.g., *hard* for shuāi 摔). While the numbers were too small to allow for a statistical comparison, it is noteworthy that the bilinguals used more different modifying words and used modified-*throw* more often than the monolinguals.

### English: Forced choice

**Figure 1.**
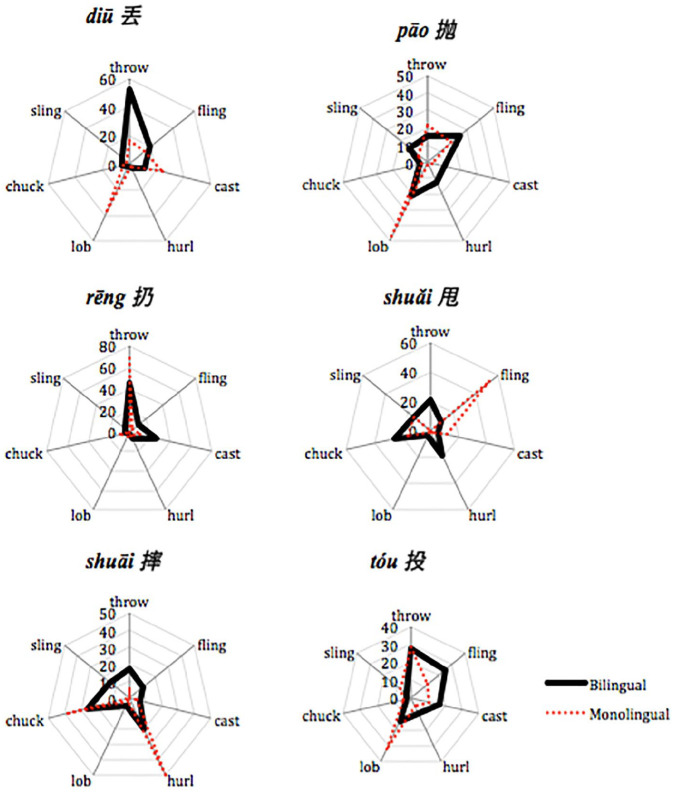
Percentage of participants who chose each English option for each typical Mandarin throwing action.

Figure 1 summarizes the responses of the participants to each of the actions (labeled by the target Mandarin verb). The English monolinguals tended to have either one (rēng 扔 = *throw*, diū 丢 = *lob*, pāo 抛 = *lob*, shuǎi 甩 = *fling*) or two preferred choices (shuāi 摔 = *chuck* or *hurl*, tóu = *lob* or *throw*) for each action. The monolinguals’ choices were often based on similarities in the characteristics of the actions. For example, as can be seen in Table 1, shuāi 摔 and *hurl* differ only on the vertical direction of the action (with shuāi 摔 being downward and *hurl* upward). The verb *lob* was not included in the Wang and Gao (2016) study so it is not clear why monolinguals so often chose *lob* for pāo 抛 and t*óu* 投.

In contrast to the monolinguals, the bilinguals’ choices tended to be more spread out across the other options. Nonetheless, the bilinguals’ most frequent choice differed from monolinguals only for rēng 扔, χ^2^ (*N* = 73, *df* = 1) = 3.93, *p* < .05, and for diū 丢, χ^2^ (*N* = 73, *df* = 1) = 10.04, *p* < .01. As can be seen in Figure 1, for rēng 扔, the bilinguals were more likely to choose *cast* more than the monolinguals. For diū 丢, the bilinguals mostly chose *throw* while the monolinguals mostly chose *lob*. Like the monolinguals, the bilinguals’ choices were often similar on many of the characteristics summarized in Table 1. For example, the bilinguals often chose *chuck* for shuǎi 甩, a choice that differs from shuǎi 甩 only on the vertical direction of the motion: *chuck* is often upward and shuǎi 甩 downward.

**Figure 2.**
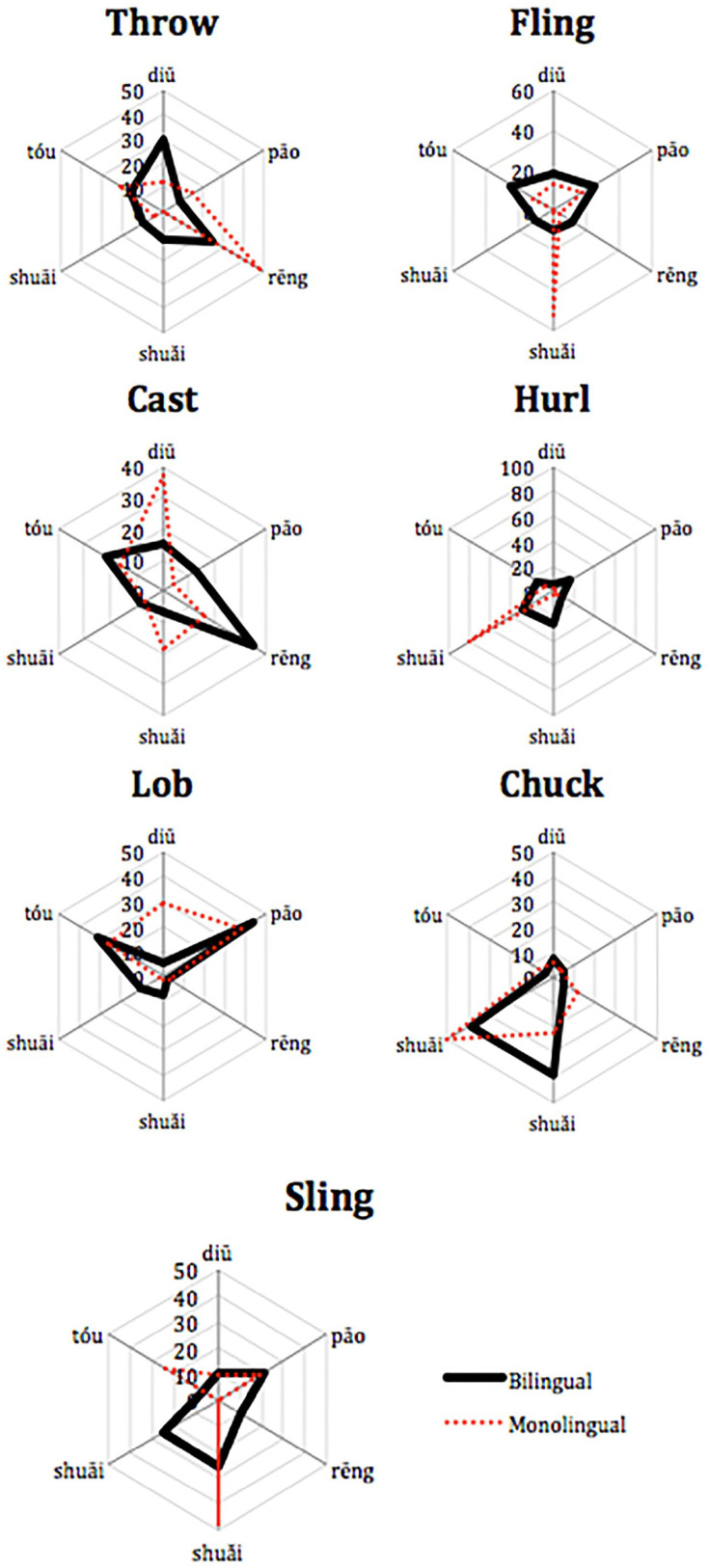
For each English option, percentage of participants who chose each typical Mandarin throwing action.

As can be seen in Figure 1, the most frequent choice of participants does not always correspond to a large percentage of participants (e.g., see the bilinguals for shuāi 摔). Therefore, it is also interesting to see how frequently the participants chose a particular Mandarin throwing action as the appropriate choice for each English word. Figure 2 summarizes the choices of the participants corresponding to particular Mandarin throwing actions. The English monolinguals tended to have either one (*throw* = rēng 扔, *fling* = shuǎI 甩, *cast* = diū 丢, *hurl* = shuāi 摔, and *sling* = shuǎi 甩) or two preferred choices (*chuck* = shuāi 摔 or shuǎi 甩). *Lob* was fairly equally distributed across tóu 投, diū 丢, and pāo 抛. In contrast, the bilinguals’ choices tended to be more spread out across the other options, with two notable exceptions. For *chuck*, like the monolinguals, both shuāi 摔 or shuǎi 甩 were associated about equally often by bilinguals. Nonetheless, the bilinguals’ most frequent choice differed from monolinguals only for *throw*, χ^2^ (*N* = 73, *df* = 1) = 10.04, *p* < .01, *hurl*, χ^2^ (*N* = 73, *df* = 1) = 7.30, *p* < .01, and *lob*, χ^2^ (*N* = 73, *df* = 1) = 5.42, *p* < .02. As can be seen in Figure 2, for *throw*, the bilinguals were more likely to choose diū 丢 more than the monolinguals. For *hurl*, the bilinguals had no clear association, while the monolinguals mostly associated shuāi 摔. Finally, for *lob*, the bilinguals associated tóu 投 and pāo 抛 about equally often (and almost never diū 丢, like the English monolinguals).

**Figure 3.**
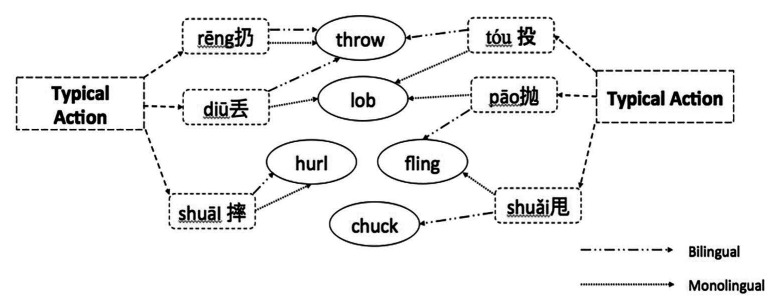
Most frequent answers on English forced-choice task – typical actions.

**Figure 4.**
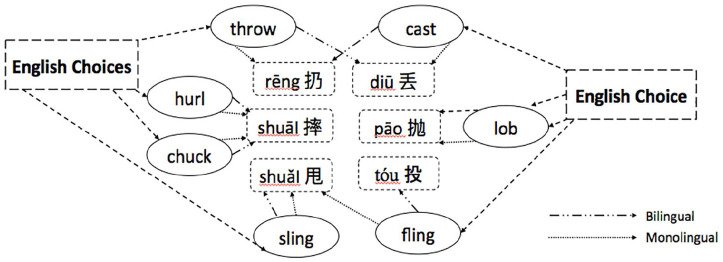
Most frequent answers on English forced-choice task – English choices.

Figures 3 and 4 summarize the results in terms of the most frequent responses of the bilinguals and monolinguals. As can be seen in Figure 3, bilinguals did choose some of the same most frequent English responses as monolinguals (for rēng 扔, diū 丢, and shuāi 摔). The bilinguals also chose different most frequent English responses from the monolinguals (for tóu 投, pāo 抛, and shuǎI 甩). Similarly, Figure 4 shows that bilinguals often chose the same most frequent action as monolinguals for *hurl, chuck, sling*, and *lob*. However, they showed differences on the most frequent chosen action for *throw, cast*, and *fling*.

## Discussion

The purpose of this study was to identify the strategies bilinguals used to label typical Mandarin throwing actions in English. The Mandarin verbs have few straightforward translations into English (Wang & Gao, 2016). In other words, for most of the *throw* verbs, there is at best partial overlap between the concepts underlying the Mandarin and English verbs. We asked Mandarin-English sequential bilinguals and English monolinguals to label and to select words in English corresponding to typical Mandarin throwing actions. The English monolinguals in this study showed a high degree of agreement on a choice of usually either one or two English verbs to label these throwing actions. For them, *throw* corresponds to rēng 扔, *lob* to diū 丢, pāo 抛, and tóu 投, *fling* to shuǎi 甩, and either *chuck* or *hurl* shuāi 摔 or shuǎi 甩 (see Figures 3 and 4).

If the Mandarin-English bilinguals were conceptualizing English words in the target language way, they should not differ from the monolinguals. Indeed, the bilinguals showed a good deal of agreement with the monolinguals for some actions: *throw* most often corresponded to rēng 扔, *lob* to pāo 抛, and either *chuck* or *hurl* to shuāI 摔. There were, however, some interesting ways in which the bilinguals’ lexical selections differed from the monolinguals’. Firstly, the bilinguals tended to show less homogeneity than the monolinguals in associating an action and an English word (Figures 1 and 2). Secondly, for diū 丢, most monolinguals chose the label *lob*, while most bilinguals avoided choosing *lob* (preferring that term for pāo 抛 or even tóu 投). The most frequent choice for diū 丢 was *throw*. The salient characteristics of *lob* for English monolinguals were not included in Wang and Gao (2016), so it is not entirely clear why participants showed this pattern of choices. One possibility is that the bilinguals were choosing the most general term for throwing. However, recall that they often chose rēng 扔 (most frequently labeled *throw* in English) for diū 丢 events in Mandarin. An added complication in the interpretation of the present study was that we failed to provide participants with the choice *toss* on the forced-choice task, the English word that would correspond the most closely to diū 丢. Nevertheless, it seems likely that the bilinguals conceptualized diū 丢 and rēng 扔 as similar enough to merit the same label in English. Bilinguals also differed notably from monolinguals for *fling*, often selecting it for tóu 投, whereas monolinguals rarely selected *fling* at all. For the bilinguals, the sideways motion of tóu 投 may have been best captured by *flin*g, the English verb that Wang and Gao (2016) found elicited the most sideways movement. One final noteworthy difference between the bilinguals and the monolinguals is that individual bilinguals gave different answers for shuǎi 甩, unlike monolinguals who often selected *chuck* or *hurl*.

Note that this discussion of the results synthesizes the bilinguals’ choices from both a labeling task and a word-choice task. There were some differences between the participants’ performance on the task, in predictable directions. For example, the participants were more likely to choose the generic *throw* in the labeling task than in the word-choice task. This result is likely due to the fact that the labeling task involves searching the mental lexicon for an appropriate label. Once the difficulty of lexical retrieval is taken into account, the results from the two tasks most often pointed to the same labels for particular actions.

The results do not correspond neatly to the prediction that bilinguals adopt either target-language concepts or L1 concepts (i.e., differentiation of their two languages; cf. Pavlenko, 2010). The bilinguals in the present study showed some signs of thinking for speaking English but also some differences from monolinguals. These differences can sometimes be attributed to the concepts underlying their Mandarin verbs. For example, the bilinguals showed some overlap in labeling rēng 扔 and diū 丢 in Mandarin and showed the same overlap in English. Pavlenko (2002) reported similar results for Russian-English bilinguals speaking about emotions.

The results also do not correspond neatly to the prediction that the bilinguals’ responses would be related to the degree of overlap in the concepts across languages (Pavlenko, 2008). There is a fair bit of overlap for rēng 扔 and *throw* and diū 丢 and *toss* (Wang & Gao, 2016). We predicted that bilinguals might show greater homogeneity in their responses to those actions in English than the other actions, where the concepts are only partially equivalent. They did not.

The results of the present study are consistent with those of Ibarretxe-Antuñano et al. (2016). In that study, bilinguals generated many more different verbs than native speakers to refer to actions of placing objects. The bilinguals also sometimes added words to label characteristics that differed across their two languages (such as intentionality or force dynamics). A recent study conducted by Gao (2020) on Mandarin-English bilingual speakers’ conceptualization of force and motion in the semantics of *pull* action verbs (e.g., bá 拔, lā 拉, tuō 拖, zhuāi 拽) showed that bilingual speakers tended to make more efforts in trying to differentiate the action differences and, as a result, they used more verbs than the typical *pull* verbs given to describe the actions. Similarly, in the present study, most often, the bilinguals came up with different solutions across individuals and across Mandarin actions to refer to typical Mandarin throw actions. In labeling, the bilinguals sometimes modified *throw* to highlight the distinctions between throw actions in terms of directionality and force (see Table 4), the distinctions that guide verb choice in Mandarin (Wang & Gao, 2016). In the forced-choice task, unlike the monolinguals, the bilinguals often seemed to assume that there was one English verb that corresponded to each Mandarin action, but often chose different corresponding verbs across participants (see Figures 1 and 2).

The above results accord with our observation and experiences in teaching classes with both native and bilingual speakers of English participating in class discussions. Bilingual speakers tended to use more varieties of words or showed hesitations in their choices of words when they were unsure of whether they were using certain words correctly. This could be due to the language differences between Chinese and English, as Tardif et al. (1999) pointed out that the Chinese language has more specific action verbs that allow Mandarin speakers to acquire specific action verbs at an earlier age. Also, there could be individual differences in bilinguals’ use of strategies. One important implication of the results of this study (along with those of Ibarretxe-Antuñano et al., 2016) for the relationship of language and thought is the individual differences in strategy choice influenced by one’s mother tongue. Future studies can focus on explanations for these types of individual differences. Previous studies have shown that a number of variables affect how bilinguals think for speaking in their languages, including age of acquisition, amount of exposure, and proficiency (Malt et al., 2015; Pavlenko & Malt, 2011). Furthermore, the choice of strategies is likely a dynamic ever-changing process (Hernandez et al., 2005; Malt et al., 2015). Future studies can also go beyond single word studies (as recommended by Ervin-Tripp, 2000). Future studies could also test whether bilinguals’ concepts can change with training: one study showed that L2 naming patterns emerged after relatively short training sessions in the lab (Malt et al., 2016).

There were a number of limitations to the present study that can be addressed in future studies. In this study, some of the analyses were suspect because the number of items included fell below the number required for medium statistical power. Future studies can include more participants to address this issue. Similarly, only six throwing actions were included in this study. The inclusion of such few items meant that we did not have the statistical power to explore some intriguing responses. Notably, both the monolinguals and the bilinguals sometimes used modifiers to describe the actions, but they used them too infrequently to allow us to test when they were used. Also, we understand that the choice of a particular *throw* near-synonym can be affected by the object thrown. For instance, it would be hard to *lob* a frisbee or an unfolded handkerchief. In this study, we used only one object across all items. Future studies can test how the choice of the object relates to verb choice. Finally, the research design could also be improved in future studies by taking both Mandarin and English throw actions as the baselines for the preparation of the action video clips as stimuli for action matching tests.

One particularly important future direction for understanding the individual differences in bilinguals’ thinking for speaking of near-synonyms in their L2 is collecting information about individuals’ learning history. L2 learners often learn near-synonyms in the context of collocations (Ahmadian & Darabi, 2012). This kind of incidental learning has been shown to be ineffective in learning specific meanings of words in a L2 (De Groot, 2011). Learning the meaning of words in context could also result in a great deal of individual interpretations as to what near-synonyms might mean.

In conclusion, this study has shown that bilinguals used a variety of different strategies to refer to the not-easily-translatable in their L2. Further studies will be necessary to uncover exactly when particular strategies are used.

## Appendix

**Appendix.** Number of labels for typical Mandarin throwing actions. (A) Mandarin-English bilinguals in Mandarin.

	rēng 扔	diū 丢	pāo 抛	shuǎi 甩	shuāi 摔	tóu 投
rēng 扔	15	14	1	4	1	8
rēng 扔 +	0	1	0	2	3	1
diū 丢	4	4	0	8	1	4
pāo 抛	4	12	29	1	0	7
shuǎi 甩	0	1	0	8	3	0
shuāi 摔	0	0	0	5	15	0
tóu 投	9	2	3	1	0	14
Other	2	0	0	5	11	0

**Table:** (B) English monolinguals in English.

	rēng 扔	diū 丢	pāo 抛	shuǎi 甩	shuāi 摔	tóu 投
Throw	26	5	2	13	8	11
Throw +	1	0	1	0	1	1
Toss	3	24	22	4	1	5
Fling	0	0	0	2	0	1
Cast	0	0	0	0	0	0
Hurl	0	0	0	0	1	0
Lob	0	1	4	0	0	4
Chuck	1	0	0	5	5	0
Sling	0	0	0	0	0	0
Other	3	3	5	9	18	12

**Table:** (C) Mandarin-English bilinguals in English.

	rēng 扔	diū 丢	pāo 抛	shuǎi 甩	shuāi 摔	tóu 投
Throw	25	24	20	19	9	17
Throw +	4	2	3	4	2	2
Toss	0	2	5	2	1	4
Fling	1	1	2	2	1	3
Cast	4	1	2	0	0	2
Hurl	0	0	0	0	1	0
Lob	0	0	4	1	1	0
Chuck	0	0	1	1	1	0
Sling	0	0	0	1	1	0
Other	10	12	6	14	17	14

## References

[bibr1-13670069211022853] Ahmadian M. Darabi A. (2012). A study of the relationship between EFL learners’ knowledge of near synonyms and their collocational behaviour. Journal of Educational and Social Research, 2, 179–188.

[bibr2-13670069211022853] Alvarado N. Jameson K. A. (2011). Shared knowledge about emotion among Vietnamese and English bilingual and monolingual speakers. Journal of Cross-Cultural Psychology, 42, 963–982.

[bibr3-13670069211022853] Ameel E. Storms G. Malt B. Sloman S. (2005). How bilinguals solve the naming problem. Journal of Memory and Language, 52, 309–329.

[bibr4-13670069211022853] Apresjan V. (1997). Emotion metaphors and cross-linguistic conceptualization of emotions. Cuadernos de filologia inglesa, 6, 179–195.

[bibr5-13670069211022853] Athanasopoulos P. (2009). Cognitive representation of colour in bilinguals: The case of Greek blues. Bilingualism: Language and Cognition, 12, 83–95.

[bibr6-13670069211022853] Aveledo F. Athanasopoulos P. (2016). Second language influence on first language motion event encoding and categorization in Spanish-speaking children learning L2 English. International Journal of Bilingualism, 20, 403–420.

[bibr7-13670069211022853] Bender A. Beller S. Klauer K. C. (2011). Grammatical gender in German: A case for linguistic relativity? Quarterly Journal of Experimental Psychology, 64, 1821–1835. 10.1080/17470218.2011.582128 21740112

[bibr8-13670069211022853] Barbosa P. Nicoladis E. Keith M. (2017). Bilingual children's lexical strategies in a narrative task. Journal of Child Language, 44, 829–849. 27238581 10.1017/S030500091600026X

[bibr9-13670069211022853] Cadierno T. Ruiz L. (2006). Motion events in Spanish L2 acquisition. Annual Review of Cognitive Linguistics, 4, 183–216.

[bibr10-13670069211022853] Casasanto D. (2016). A shared mechanism of linguistic, cultural, and bodily relativity. Language Learning, 66, 714–730.

[bibr11-13670069211022853] Chen J. Shirai Y. (2010). The development of aspectual marking in child Mandarin Chinese. Applied Psycholinguistics, 31, 1–28.

[bibr12-13670069211022853] De Groot A. M. B . (2011). Language and cognition in bilinguals and multilinguals: An introduction. New York, NY: Psychology Press.

[bibr13-13670069211022853] De Jonge B . (1993). The existence of synonyms in a language: Two forms but one, or rather two, meanings? Linguistics, 31, 521–538.

[bibr14-13670069211022853] Divjak D. (2006). Ways of intending: Delineating and structuring near-synonyms. Trends in Linguistics Studies and Monographs, 172, 19.

[bibr15-13670069211022853] Ervin-Tripp S. (2000). Bilingual minds. Bilingualism: Language and Cognition, 3, 10–12.

[bibr16-13670069211022853] Filipović L. (2013). Constructing causation in language and memory: Implications for access to justice in multilingual interactions. International Journal of Speech, Language and the Law, 20, 1–19.

[bibr17-13670069211022853] Gao H. (2001). The physical foundation of the patterning of physical action verbs: A study of Chinese verbs. Lund, Sweden: Lund University Press.

[bibr18-13670069211022853] Gao H. H. (2008, April 18). The transferability of lexical properties in bilinguals’ application of Chinese classifiers [Paper presentation]. Workshop of the Transferability of Lexical Properties between Two Languages in Bilinguals. NTU, Singapore.

[bibr19-13670069211022853] Gao H. H. (2010). Computational lexicography: A feature-based approach in designing an e-dictionary of Chinese classifiers. In Proceedings of the 2nd SIGLEX Endorsed Workshop on Cognitive Aspects of the Lexicon (COLING 2010) (pp. 56–65). Beijing, China.

[bibr20-13670069211022853] Gao H. H. (2015). Association of nouns and classifiers by bilingual children in Mandarin Chinese. In Airenti G. Bara B. G. Sandini G. (Eds.), Proceedings of the EuroAsianPacific Joint Conference on Cognitive Science (pp. 566–571). Torino, Italy.

[bibr21-13670069211022853] Gao H. H. (2020). Mandarin speakers’ conceptualization of force and motion in the semantics of pull verbs of hand action in Mandarin Chinese. In Su Q. Zhan W. (Eds.), From minimal contrast to meaning construct. Frontiers in Chinese linguistics (Vol. 9, pp. 63–65). Singapore: Springer.

[bibr22-13670069211022853] Gao H. H. Wang H. S. Nicoladis E. (2016). The delineation of throw verbs in Chinese: A behavioral and perceptive approach. Journal of Cognitive Science, 17, 95–131.

[bibr23-13670069211022853] Green D. W. (1998). Bilingualism and thought. Psychologica Belgica, 38, 251–276.

[bibr24-13670069211022853] Gullberg M. (2011). Language-specific encoding of placement events in gestures. In Bohnemeyer J. Pederson E. (Eds.), Event representation in language and cognition (pp. 166–188). Cambridge, MA: Cambridge University Press.

[bibr25-13670069211022853] Hernandez A. Li P. MacWhinney B. (2005). The emergence of competing modules in bilingualism. Trends in Cognitive Sciences, 9, 220–225. 15866148 10.1016/j.tics.2005.03.003 PMC4107303

[bibr26-13670069211022853] Hohenstein J. Eisenberg A. Naigles L. (2006). Is he floating across or crossing afloat? Cross-influence of L1 and L2 in Spanish-English bilingual adults. Bilingualism: Language and Cognition, 9, 249–261.

[bibr27-13670069211022853] Ibarretxe-Antuñano I. (2012). Placement and removal events in Basque and Spanish. In Kopecka A. Narasimhan B. (Eds.), Events of putting and taking: A crosslinguistic perspective (pp. 123–143). Amsterdam: John Benjamins.

[bibr28-13670069211022853] Ibarretxe-Antuñano I. Cadierno T. Hijazo-Gascón A. (2016). The role of force dynamics and intentionality in the reconstruction of L2 verb meanings: A Danish-Spanish bidirectional study. Review of Cognitive Linguistics, 14, 136–160.

[bibr29-13670069211022853] January D. Kako E. (2007). Re-evaluating evidence for linguistic relativity: Reply to Boroditsky (2001). Cognition, 104, 417–426. 16914131 10.1016/j.cognition.2006.07.008

[bibr30-13670069211022853] Lee O. Fradd S. H. Sutman F. X. (1995). Science knowledge and cognitive strategy use among culturally and linguistically diverse students. Journal of Research in Science Teaching, 32, 797–816.

[bibr31-13670069211022853] Li P. Gleitman L. (2002). Turning the tables: Language and spatial reasoning. Cognition, 83, 265–294. 10.1016/s0010-0277(02)00009-4 11934404

[bibr32-13670069211022853] Lucy J. A. (2016). Recent advances in the study of linguistic relativity in historical context: A critical assessment. Language Learning, 66, 487–515.

[bibr33-13670069211022853] Lyons J. (1995). Linguistic semantics: An introduction. Cambridge: Cambridge University Press.

[bibr34-13670069211022853] Malt B. C. Jobe R. L. Li P. Pavlenko A. Ameel E. (2016). What constrains simultaneous mastery of first and second language word use? International Journal of Bilingualism, 20, 684–699.

[bibr35-13670069211022853] Malt B. C. Li P. Pavlenko A. Zhu H. Ameel E. (2015). Bidirectional lexical interaction in late immersed Mandarin-English bilinguals. Journal of Memory and Language, 82, 86–104.

[bibr36-13670069211022853] Malt B. C. Sloman S. (2003). Linguistic diversity and object naming by non-native speakers of English. Bilingualism: Language and Cognition, 6, 47–67.

[bibr37-13670069211022853] Narasimhan B. Kopecka A. Bowerman M. Gullberg M. Majid A. (2012). Putting and taking events: A crosslinguistic perspective. In Kopecka A. Narasimhan B. (Eds.), Events of putting and taking: A crosslinguistic perspective (pp. 1–18). Amsterdam: John Benjamins.

[bibr38-13670069211022853] Matlock T. Ramscar M. Boroditsky L. (2005). On the experiential link between spatial and temporal language. Cognitive Science, 29, 655–664. 21702788 10.1207/s15516709cog0000_17

[bibr39-13670069211022853] Odlin T. (2005). Crosslinguistic influence and conceptual transfer: What are the concepts? Annual Review of Applied Linguistics, 25, 3–25.

[bibr40-13670069211022853] Panayiotou A. (2004). Bilingual emotions: The untranslatable self. Sociolinguistic Studies, 5, 1–19.

[bibr41-13670069211022853] Pavlenko A. (2002). Bilingualism and emotions. Multilingua, 21, 45–78.

[bibr42-13670069211022853] Pavlenko A. (2008). Emotion and emotion-laden words in the bilingual lexicon. Bilingualism: Language and cognition, 11, 147–164.

[bibr43-13670069211022853] Pavlenko A. (2009). Conceptual representation in the bilingual lexicon and second language vocabulary learning. In Pavlenko A. (Ed.), The bilingual mental lexicon: Interdisciplinary approaches (pp. 125–160). Clevedon, England: Multilingual Matters.

[bibr44-13670069211022853] Pavlenko A. (2010). Verbs of motion in L1 Russian of Russian–English bilinguals. Bilingualism: Language and Cognition, 13, 49–62.

[bibr45-13670069211022853] Pavlenko A. (2014). The bilingual mind. Cambridge: Cambridge University Press.

[bibr46-13670069211022853] Pavlenko A. Driagina V. (2007). Russian emotion vocabulary in American learners’ narratives. The Modern Language Journal, 91, 213–234.

[bibr47-13670069211022853] Pavlenko A. Malt B. C. (2011). Kitchen Russian: Cross-linguistic differences and first-language object naming by Russian–English bilinguals. Bilingualism: Language and Cognition, 14(1), 19–45.

[bibr48-13670069211022853] Pavlenko A. Volynsky M. (2015). Motion encoding in Russian and English: Moving beyond Talmy’s typology. The Modern Language Journal, 99(S1), 32–48.

[bibr49-13670069211022853] Quek S. L. Gao H. H. (2011). An experimental investigation of the cognitive basis of Malaysian Chinese speakers’ association of one noun with multiple classifiers. In Proceedings of the 12th Chinese Lexical Semantics Workshop (pp. 232–243). Taipei, Taiwan: Crane Publishing. (in Chinese).

[bibr50-13670069211022853] Roberson D. Davidoff J. Davies I. R. L. Shapiro L. (2005). Cognitive representation of colour in bilinguals: The case of Greek blues. Cognitive Psychology, 50, 378–411. 15893525 10.1016/j.cogpsych.2004.10.001

[bibr51-13670069211022853] Samuel S. Cole G. Eacott M. J. (2019). Grammatical gender and linguistic relativity: A systematic review. Psychonomic bulletin & review, 26(6), 1767–1786. 10.3758/s13423-019-01652-3 31429058

[bibr52-13670069211022853] Slobin D. I. (1996). Two ways to travel: Verbs of motion in English and Spanish. In Shibatani M. Thompson S. (Eds.), Grammatical constructions: Their form and meaning (pp. 195–219). Oxford: Oxford University Press.

[bibr53-13670069211022853] Slobin D. I. Hoiting N. (1994). Reference to movement in spoken and signed languages: Typological considerations. Proceedings of the Berkeley Linguistics Society, 20, 487–505.

[bibr54-13670069211022853] Sugaya N. Shirai Y. (2009). Can L2 learners productively use Japanese tense-aspect markers? A usage-based approach. In Corrigan R. Moravcsik E. Ouali H. Wheatley K. (Eds.), Formulaic language: Vol. 2. Acquisition, loss, psychological reality, functional applications (pp. 423–444). Amsterdam: John Benjamins.

[bibr55-13670069211022853] Talmy L. (1985). Lexicalization patterns: Semantic structure in lexical forms. In Shopen T. (Ed.), Language typology and semantic description. Vol. 3: Grammatical categories and the lexicon (pp. 36–149). Cambridge: Cambridge University Press.

[bibr56-13670069211022853] Tardif T. Gelman S. A. Xu F . (1999). Putting the “noun bias” in context: a comparison of English and Mandarin. Child Development, 70, 620–635.

[bibr57-13670069211022853] Van Hell J. G. De Groot A. M . (1998). Conceptual representation in bilingual memory: Effects of concreteness and cognate status in word association. Bilingualism: Language and Cognition, 1, 193–211.

[bibr58-13670069211022853] Vigliocco G. Vinson D. P. Paganelli F. Dworzynski K. (2005). Grammatical gender effects on cognition: implications for language learning and language use. Journal of Experimental Psychology: General, 134, 501–520. 10.1037/0096-3445.134.4.501 16316288

[bibr59-13670069211022853] Wang H. Gao H. H. (2016). Cross-linguistic categorization of throwing events: A behavioral approach. Cognitive Linguistic Studies, 3, 259–276.

[bibr60-13670069211022853] Whorf B. L. (1956). Language, thought, and reality, edited by Carroll J. B. Cambridge, MA: The MIT Press.

